# *In Vitro* Reconstruction of Neuronal Networks Derived from Human iPS Cells Using Microfabricated Devices

**DOI:** 10.1371/journal.pone.0148559

**Published:** 2016-02-05

**Authors:** Yuzo Takayama, Yasuyuki S. Kida

**Affiliations:** Biotechnology Research Institute for Drug Discovery, National Institute of Advanced Industrial Science and Technology (AIST), Tsukuba, Ibaraki, Japan; Hertie Institute for Clinical Brain Research, University of Tuebingen., GERMANY

## Abstract

Morphology and function of the nervous system is maintained via well-coordinated processes both in central and peripheral nervous tissues, which govern the homeostasis of organs/tissues. Impairments of the nervous system induce neuronal disorders such as peripheral neuropathy or cardiac arrhythmia. Although further investigation is warranted to reveal the molecular mechanisms of progression in such diseases, appropriate model systems mimicking the patient-specific communication between neurons and organs are not established yet. In this study, we reconstructed the neuronal network *in vitro* either between neurons of the human induced pluripotent stem (iPS) cell derived peripheral nervous system (PNS) and central nervous system (CNS), or between PNS neurons and cardiac cells in a morphologically and functionally compartmentalized manner. Networks were constructed in photolithographically microfabricated devices with two culture compartments connected by 20 microtunnels. We confirmed that PNS and CNS neurons connected via synapses and formed a network. Additionally, calcium-imaging experiments showed that the bundles originating from the PNS neurons were functionally active and responded reproducibly to external stimuli. Next, we confirmed that CNS neurons showed an increase in calcium activity during electrical stimulation of networked bundles from PNS neurons in order to demonstrate the formation of functional cell-cell interactions. We also confirmed the formation of synapses between PNS neurons and mature cardiac cells. These results indicate that compartmentalized culture devices are promising tools for reconstructing network-wide connections between PNS neurons and various organs, and might help to understand patient-specific molecular and functional mechanisms under normal and pathological conditions.

## Introduction

The nervous system consists of the central and peripheral systems that are connected with each other, and thus form an electrical signaling network throughout the body. Although each neuron type is differentiated from different stem/progenitor cell pools, interactions between various cell types are well-coordinated both morphologically and functionally. The peripheral nervous system (PNS) is connected to the central nervous system (CNS), and this functional system is responsible for the homeostasis of various tissues and organs. Indeed, peripheral neuropathies caused by genetic disorders [1], autoimmune diseases [2], or diabetes [3,4] induce functional abnormalities in the entire body. Owing to the complexity of causes and symptoms, peripheral neuropathy is usually treated with symptomatic approaches such as surgical intervention or pain management. Therefore, understanding the molecular mechanism of peripheral neuropathy progression and the interaction of the PNS with target organs might contribute to the development of novel therapeutic methods aiming for a complete cure.

Co-culture systems can be used to model inter-organ communications *in vitro*. In the most simple co-culture method, different types of cells are mixed and co-cultured in a dish. Here, interactions are used to improve cell culture conditions or to support cell growth. For example, embryonic stem (ES) cells or induced pluripotent stem (iPS) cells are co-cultured with feeder cells, and primary neurons are co-cultured with astrocytes. Furthermore, for studying cell invasion or paracrine interactions, non-contacting co-culture conditions are required. In these cases, permeable co-culture systems have been used [5,6]. However, these conventional co-culture methods are not suitable for neuronal co-cultures, because neurons extend their neurites over a long distance, and establish connections with other types of cells. Furthermore, neuronal functions by the neurite connections are based on the parallel and distributed computations of network-wide activities [7,8]. Thus, cultured neurons and target organs have to be arranged correctly for reconstructing inter-organ neuronal networks *in vitro*.

Microfabrication techniques for preparing specific dishes are a promising way to overcome these problems in neuronal cultures. In 1977, R. B. Campenot developed a Teflon-based culture chamber to separate neurites from cell bodies [9]. The Campenot chamber, which enables a compartmentalized culturing technique, was primarily used for studying the local effects of growth factors in neurites. However, due to its size, the Campenot chamber is not suitable for culturing any neurons, and the preparation of the chamber requires practice [10]. Compared to Teflon, polydimethylsiloxane (PDMS) is a more suitable material for engineering cell culture devices, because it is biocompatible, optically transparent, and even submicron features are easy to fabricate [11]. We and others have used microfabricated PDMS chambers for the network-wide integration of rodent CNS neurons, sympathetic neurons, and cardiomyocytes [12–14]. However, these studies largely relied on animal cells because of difficulties in obtaining and handling patient-derived cells. Therefore, the construction of co-culture networks using PNS neurons, CNS neurons, or other cell types derived from human pluripotent stem cells (PSCs) could become a promising *in vitro* model system for studying peripheral neuron-related diseases.

In this study, we created co-culture networks using human PNS and CNS neurons. First, we fabricated a PDMS-based co-culture chamber, which consisted of two culture compartments connected with 20 microtunnels, and we cultured induced PNS and CNS neurons differentiated from human iPS cells. Development of their connections was evaluated with microscopic observations, immunochemical analysis, and calcium imaging. Furthermore, we prepared a co-culture system using PNS neurons and cardiomyocytes, both derived from the same human iPS cells, to confirm that our microfabricated device can be used with various cell types.

## Materials and Methods

### Ethnic statement

The use of human iPS cells was approved by the Ethics Committee of National Institute of Advanced Industrial Science and Technology (AIST).

### Device fabrication

The co-culture device was fabricated from PDMS using soft lithography and replica molding technique. For producing the master mold, SU-8 3005 (Microchem) was spin-coated on a φ76 silicon wafer (Matsuzaki Seisakusyo., Ltd.) at 4000 rpm for 60 s to reach a height of 5 μm. The coated wafer was pre-baked at 95°C for 3 min. Then, the wafer was exposed to ultraviolet (UV) light with a UV crosslinker (CL-1000L; UVP) through a custom-made photomask. The photomask was designed to fabricate 20 microtunnels with a width of 50 μm and a length of 3 mm. After UV exposure, the wafer was developed with the SU-8 developer (Microchem), and then it was rinsed with 2-propanol (Wako Pure Chemical Industries). After its development, the wafer was placed in a conventional culture dish (φ100 mm; Corning).

Mixture of the PDMS-prepolymer and curing catalyst (10:1 weight ratio; Silpot 184, Dow Corning) was poured over the fabricated wafer to achieve a thickness of 5 mm. Then, PDMS was cured in an oven at 70°C for 1h. After curing, the PDMS sheet was trimmed using a surgical knife and was released from the master. To prepare the two culture compartments, which were connected by the microtunnel structures, holes were opened with a punch (φ8 mm; Harris Uni-Core; Ted Pella). We verified that each microtunnel was at least 1 mm in length; lengths ≥450 μm have been reported to allow only axons to pass through microtunnels [15]. The PDMS chamber was sealed with a cell culture dish (φ35 mm; Corning), which was previously coated with 20 μg/ml poly-L-ornithine (PLO; Aldrich). Then 5 μg/ml laminin (Sigma-Aldrich) was poured into the PDMS chamber and was incubated in the chamber until cell plating.

### Cell culture

Human iPS cells (201B7 line) were provided by the RIKEN Bioresource Center in Japan. iPS cells were plated on culture plates coated with Laminin-511-E8 (iMatrix511, Nippi) and were maintained in mTeSR1 WO 2ME/MV medium (Stemcell Technologies) at 37°C in a 5% CO_2_ incubator. The medium was changed every day. When the iPS cells were nearly confluent, iPS colonies were digested into single cells with accutase (Life Technologies), and then cells were passaged or induced as described below.

**Fig 1:**
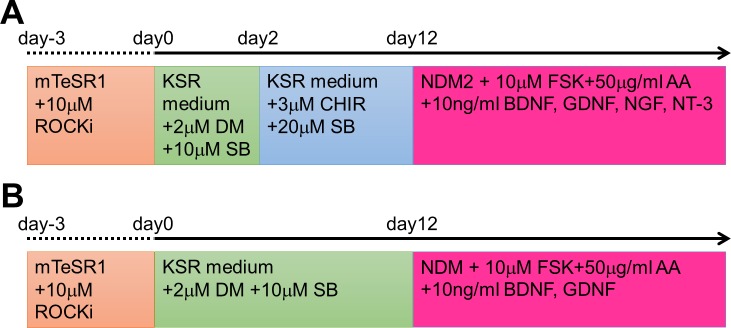
Schematic diagram showing the differentiation protocols from human iPS cells to mature neurons. Neurons of the peripheral nervous system (PNS; A) and the central nervous system (CNS; B) were induced from human induced pluripotent stem (iPS) cells with several compounds dissolved in serum-free media. Each medium and compound was added at the time indicated in (A) or (B).

Differentiation of iPS cells into CNS neurons was performed according to the following method (Fig 1A). Briefly, iPS cells were plated in 6-well plates (Corning) coated with 2-methacryloyloxyethyl phosphorylcholine (MPC) polymer (Lipidure CM5206E, NOF) at a density of 1 × 10^5^ cells/cm^2^, and cells were maintained in mTeSR1 WO 2ME/MV medium containing 10 μM Y-27632 (Wako Pure Chemical Industries). After 2–3 days, aggregated iPS cells formed embryonic bodies (EBs). Then, medium was replaced with knockout serum replacement (KSR) medium containing 2 μM dorsomorphin (DM; Sigma-Aldrich) and 10 μM SB431542 (SB; Sigma-Aldrich). The KSR medium consisted of DMEM-F12 (Wako Pure Chemical Industries), 20% KSR (Life Technologies), 1% non-essential amino acids (NEAA; Wako Pure Chemical Industries, Ltd.), 1% monothioglycerol (Wako Pure Chemical Industries), and 1% penicillin-streptomycin (P/S; Wako Pure Chemical Industries). Cell induction was performed on day 0. The KSR medium containing 2 μM DM and 10 μM SB was changed on every second day until day 12. On day 12, EBs were collected and dissociated with TrypLE (Life Technologies). Dissociated cells were plated in PLO/laminin-coated dishes at a density of 1 × 10^5^ cells/cm^2^, and cells were maintained in neural differentiation medium (NDM), which consisted of a 1:1 mixture of Neurobasal (Life Technologies) and DMEM-F12, 1% B27 supplement without vitamin A (Life Technologies), 0.5% N2 supplement (Wako Pure Chemical Industries), 1% NEAA, and 1% P/S. NDM was also supplemented with 10 μM forskolin (Wako Pure Chemical Industries), 50 μg/ml ascorbic acid (Wako Pure Chemical Industries), 10 ng/ml brain-derived neurotrophic factor (BDNF; Wako Pure Chemical Industries), and 10 ng/ml glial cell line-derived neurotrophic factor (GDNF; Wako Pure Chemical Industries). The medium was changed twice a week.

Differentiation of iPS cells into PNS neurons was performed according to the following protocol. Similar to above, the EBs of iPS cells were cultured in KSR medium containing 2 μM DM and 10 μM SB on day 0. On day 2, the medium was changed to KSR medium containing 3 μM CHIR99021 (CHIR; Cayman Chemical) and 20 μM SB. The KSR medium containing 3 μM CHIR and 20 μM SB was changed on every second day until day 12. On day 12, EBs were dissociated with TrypLE, were plated in PLO/laminin-coated dishes at a density of 1 × 10^5^ cells/cm^2^, and were maintained in NDM2, which consisted of DMEM-F12, 1% N2 supplement, 1% NEAA, and 1% P/S. NDM2 was also supplemented with 10 μM forskolin, 50 μg/ml ascorbic acid, 10 ng/ml BDNF, 10 ng/ml GDNF, 10 ng/ml nerve growth factor (NGF; Wako Pure Chemical Industries), and 10 ng/ml neurotrophin 3 (NT-3; Wako Pure Chemical Industries). The medium was changed twice a week.

For co-culture experiments in the PDMS chamber, PNS neurons were first plated into one chamber and were incubated for 30 min. After the adhesion of PNS neurons was confirmed, CNS neurons were plated into the other chamber. Each neuron type was plated at a density of 2–5 × 10^5^ cells/cm^2^. The medium in each compartment was changed on every second day.

Differentiation of iPS cells into cardiomyocytes was performed as previously described [16]. Briefly, the EBs of iPS cells were transferred into RPMI-1640 medium (Wako Pure Chemical Industries) containing 2% B27 supplement without insulin (Life Technologies) and 7.5 μM CHIR on day 0. 24 hours later, the medium was changed to fresh medium without CHIR. On day 3, the medium was changed to RPMI-1640 medium containing 2% B27 supplement without insulin and 10 μM IWR-1 (Sigma-Aldrich). On day 5, the medium was changed to fresh medium without IWR-1. From day 7, EBs were maintained in RPMI-1640 containing B27 supplement (Life Technologies), and the medium was changed on every second day. For co-cultures with PNS neurons, contracting EBs after two weeks in culture were used.

### PKH labeling

In order to differentiate neurons in the co-culture chamber, each neuron type was labeled with a PKH permanent fluorescent marker [17] before plating into the co-culture chamber. PNS and CNS neurons were labeled with 1 × 10^−6^ M PKH26 (red fluorescence; Sigma-Aldrich) and 1 × 10^−6^ M PKH67 (green fluorescence; Sigma-Aldrich), respectively. An electro multiplying (EM) charge coupled device (CCD) camera (iXon+; Andor) mounted on an inverted microscope (IX-81; Olympus) was used for detecting the fluorescence signal of PKH dyes. PKH-labeled neurons were also used for the calculation of axon growth rates. Only axonal regions that passed through microtunnels were used for the growth rate calculation in order to exclude non-linear lengths resultant from the circular shape of the culture wells. Images were visualized and analyzed with the ImageJ software (National Institutes of Health; available at http://imagej.nih.gov/ij/).

### Immunochemical staining

The cultures were rinsed with phosphate-buffered saline (PBS) and then were fixed with 3.7% formaldehyde (Wako) in PBS for 10 min at room temperature (RT). After fixation, the cells were permeabilized with PBS containing 0.2% Tween-20 (Wako Pure Chemical Industries) for 10 min, and then were blocked with PBS containing 4% Block Ace (DS Pharma Biomedical) and 0.2% Tween-20 for 1 h at RT. Primary antibodies diluted in the Can Get Signal Solution 1 (Toyobo) were incubated with the cells overnight at 4°C. Next day, the cells were washed three times with PBS containing 0.2% Tween-20, and then were incubated for 2 h at RT with the secondary antibodies (anti-mouse Alexa Fluor-488 and anti-rabbit Alexa Fluor-555; 1:1000; Life Technologies) diluted in the Can Get Signal 2 solution (Toyobo). The primary antibodies used in this study were as follows: mouse anti-class III beta-tubulin (TUJ1; 1:1000; Covance), mouse anti-P75NTR (neurotrophin receptor) (1:200; Advanced Targeting Systems), mouse anti-HNK-1 (1:500; Sigma-Aldrich), mouse anti-cTnT (cardiac Trophonin T) (1:500; Abcam), rabbit anti-Synapsin-1 (1:1000; Millipore), and rabbit anti-Peripherin (1:1000; Millipore). To stain the nuclei, 0.2 μg/ml Hoechst 33342 (Dojindo Molecular Technologies) was added to the Can Get Signal 2 solution.

### Calcium Imaging

Calcium dynamics were visualized with a calcium imaging technique. The cells were labeled with 5 μg/ml Fluo-4/AM (Invitrogen), a calcium indicator dye, for 30 min. After labeling, the medium was replaced with Ringer’s solution (148 mM NaCl, 2.8 mM KCl, 2 mM CaCl_2_, 1 mM MgCl_2_, 10 mM HEPES, and 10 mM glucose; pH 7.4). Then, the cells were placed on the stage of the inverted microscope, and fluorescence was detected with the EM CCD camera. In PNS-CNS co-culture experiments, electrical stimulation was used to evoke cellular responses. For stimulation, a pair of platinum electrodes (0.5 mm diameter; Nilaco) was immersed into the solution, and electrical pulses with a constant voltage were delivered to the cells using an electrical stimulator (SEN-3401; Nihon Kohden). Negative phase pulses with 3 ms duration and 10 V intensity were used. A frame rate of 2 frame/s was used for the PNS-CNS co-cultures. For cardiomyocytes, a frame rate of 14 frame/s was used. The recorded signals were analyzed with the ImageJ software.

## Results

### In vitro differentiation of PNS and CNS neurons from human iPS cells

**Fig 2:**
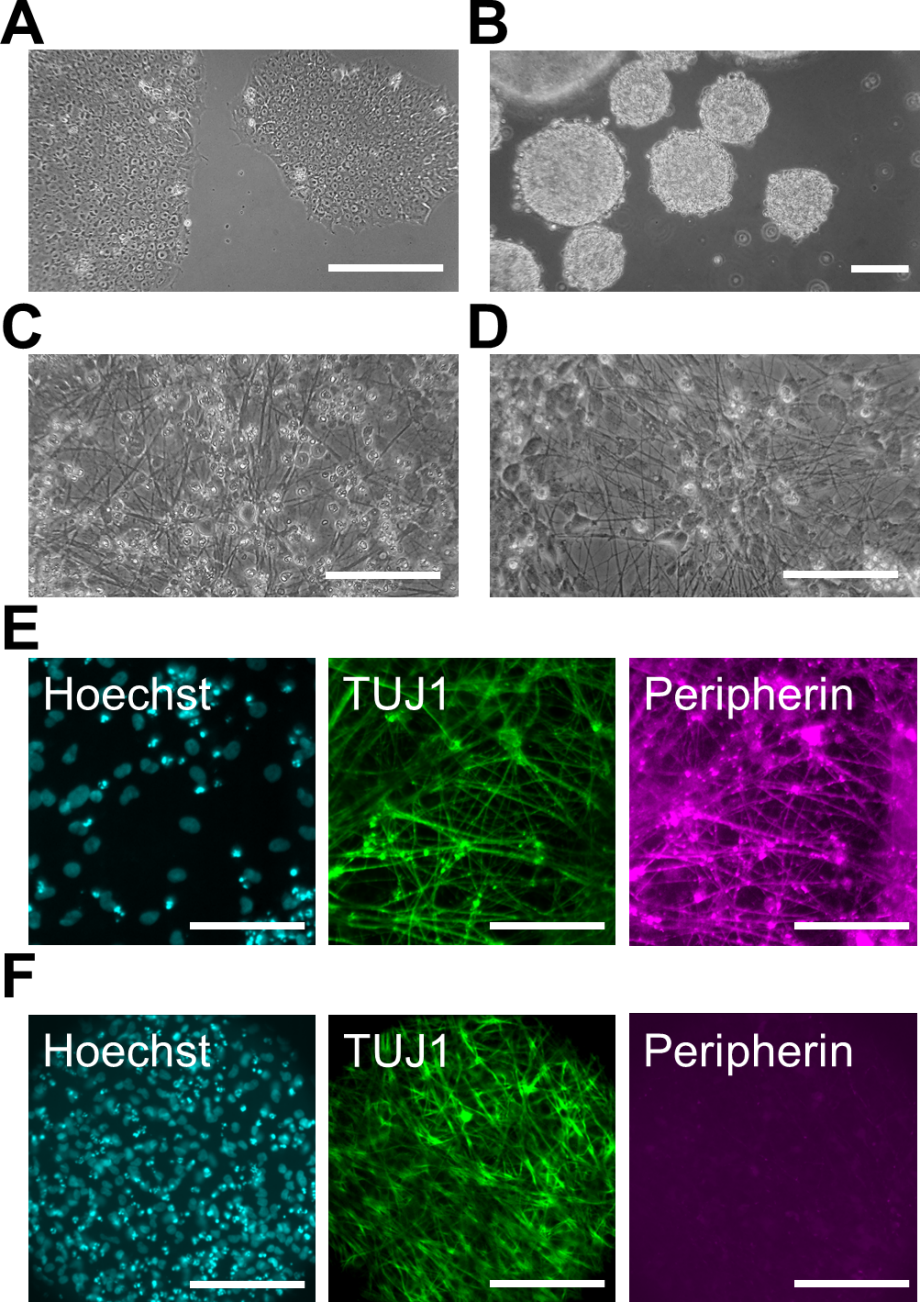
Differentiation of human PNS and CNS neurons. (A) Phase-contrast image of pre-differentiated human induced pluripotent stem (iPS) cells under a feeder-free condition. (B) Phase-contrast image of embryonic bodies (EBs) before chemical induction on day 0. (C, D) Phase-contrast image of differentiated peripheral nervous system (PNS) neurons (C) and central nervous system (CNS) neurons (D). Induced neurites were detected on day 23 (C) and day 15 (D) in PNS and CNS neurons, respectively. (E, F) Immunofluorescent labeling with antibodies specific for class III beta-tubulin (TUJ1) and Peripherin in PNS (E) and CNS neurons (F) on day 22. Cell nuclei were counterstained with Hoechst 33342. Scale bar: 100 μm.

Each neuron type was differentiated from same human iPS cells. We followed previously established protocols to differentiate human iPS cells (201B7 cell line) into human PNS and CNS neurons *in vitro* (Fig 2A). Briefly, we induced PNS neurons using neural crest cell induction with CHIR, which inhibits glycogen synthase kinase 3 beta (GSK-3β) signaling [18,19], via the formation of EBs (Fig 2B). The induced cells were re-plated in PLO/laminin-coated dishes, and we continued the differentiation *in vitro* for at least 2 weeks (Fig 2C). Most differentiated cells expressed the type III intermediate filament Peripherin, which is a marker of peripheral neurons, and TUJ1 in their neurites (Fig 2E). We also confirmed that Peripherin-positive neurons expressed the neural crest markers P75NTR and HNK-1 (S1 Fig). These data indicate that differentiated PNS neurons have characteristics of neural crest-lineage cells. On the other hand, we used a dual SMAD inhibition protocol under non-adherent conditions for the induction of CNS neurons. After 2 weeks of induction, the EBs were re-plated in PLO/laminin-coated dishes, and the cells started to extend neurites almost immediately (Fig 2D). Immunochemical staining revealed that the neurites of most cells expressed TUJ1 at a high level on day 15 (Fig 2F). However, Peripherin and P75NTR were not expressed, suggesting that these TUJ1-positive neurons were CNS neurons [20]. Thus, we prepared human PNS and CNS neurons in a large quantity *in vitro*.

### Co-culture of PNS and CNS neurons in the fabricated culture device

**Fig 3:**
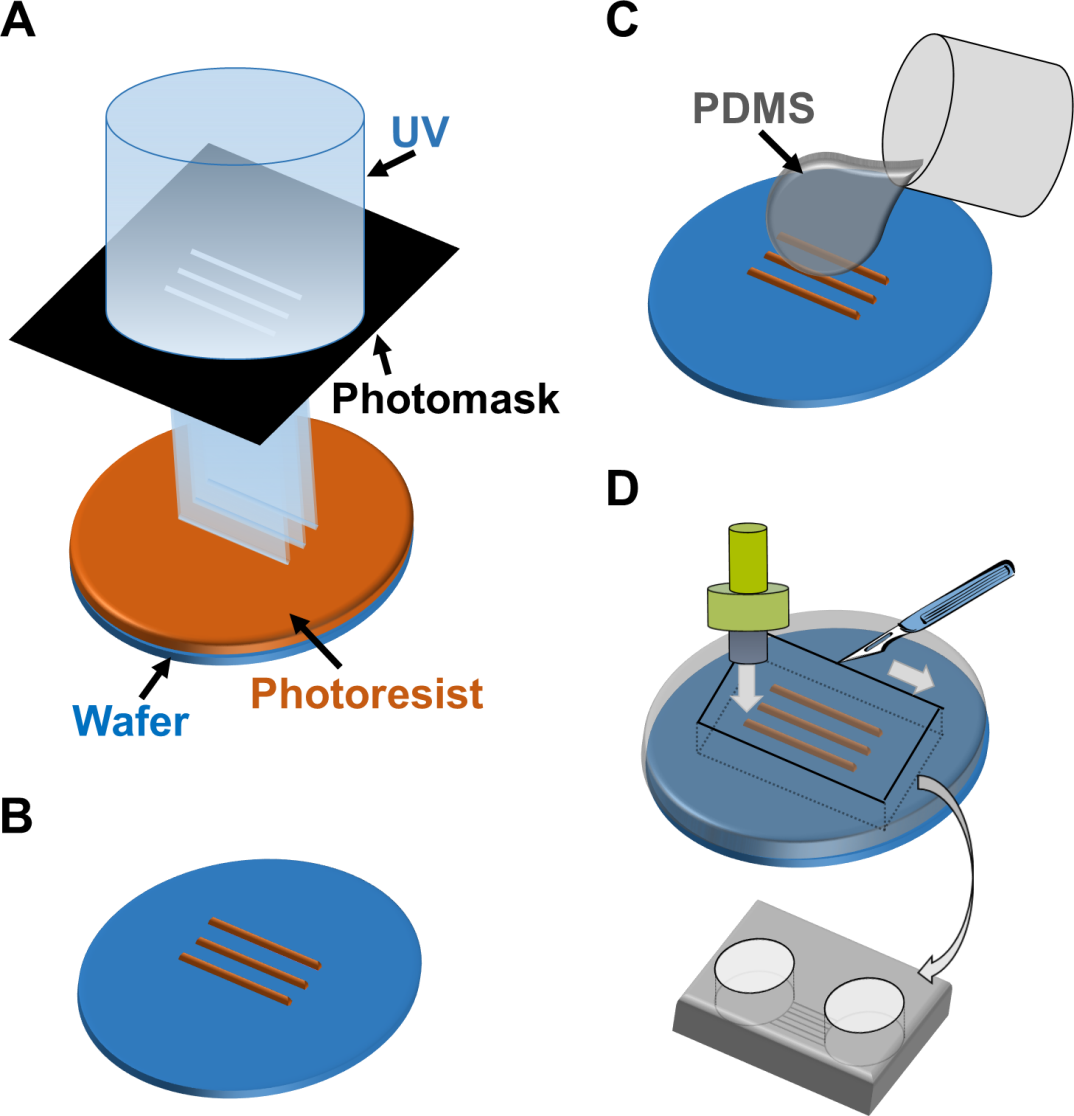
Schematic outline of the microfabrication process to manufacture the co-culturing device. (A) UV exposure of the photoresist through the photomask. (B) Development of micropatterns to fabricate the master mold. (C) Casting and curing of polydimethylsiloxane (PDMS) onto the maser mold. (D) Cutting, punching, and releasing of the PDMS chamber from the master mold.

**Fig 4:**
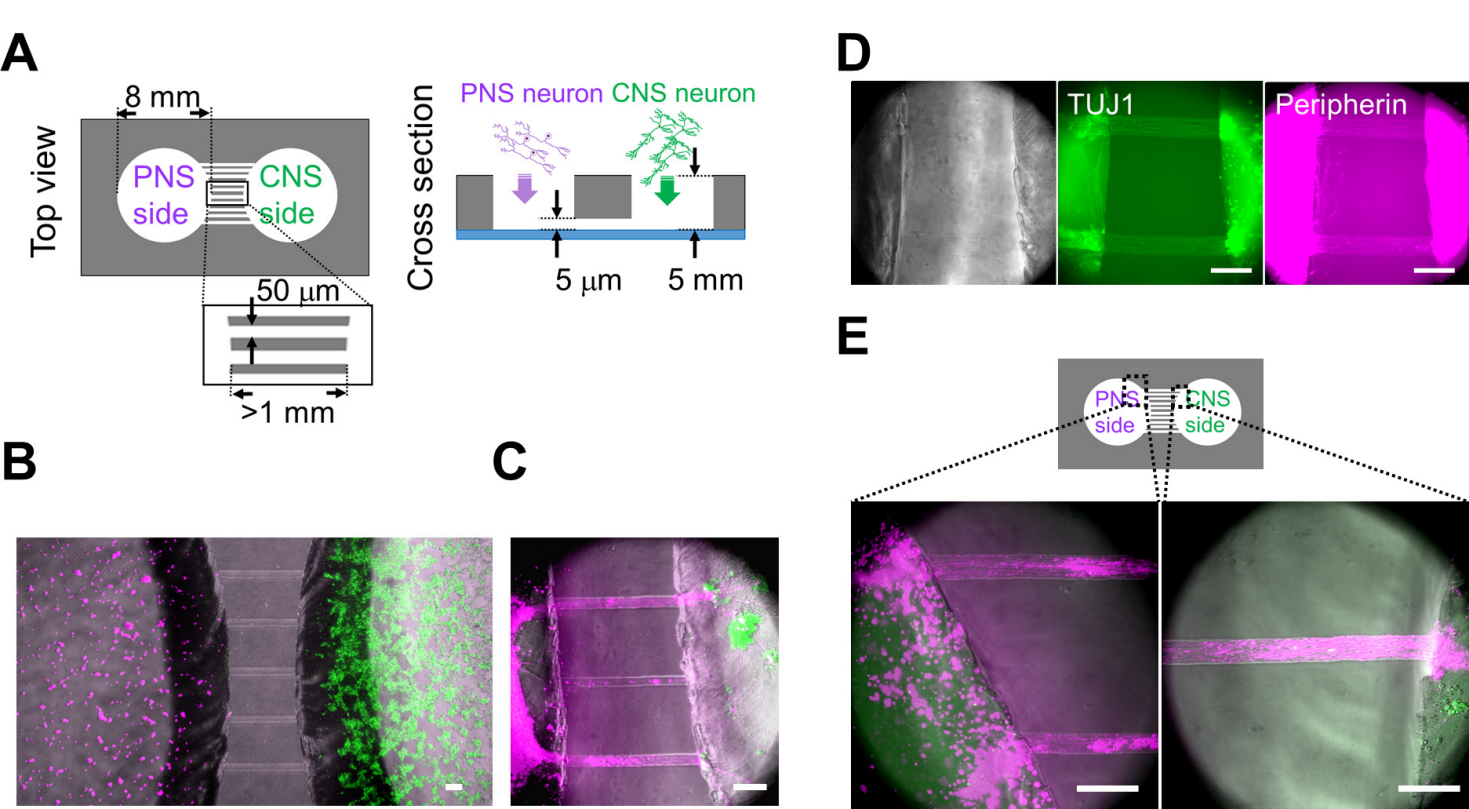
Co-culturing of PNS and CNS neurons in the PDMS chamber device. (A) Schematic diagram of the polydimethylsiloxane (PDMS) co-culture chamber. Diameter of the chambers was 8 mm. Width of the microtunnels was 50 μm. Although the length of each microtunnel varied depending on its location, the length of each microtunnel was at least 1 mm. The neurites were able to pass through the 5-μm-high microtunnels. (B) Co-cultured neurons close to the microtunnels one day after cell plating. Peripheral nervous system (PNS) neurons were labeled with PKH26 (magenta), and central nervous system (CNS) neurons were labeled with PKH67 (green). (C) Most neurites of PNS neurons passed through the microtunnels 12 days after cell plating. Bundles (magenta) originating from PNS neurons reached the aggregated CNS neurons (green). (D) Phase-contrast images and immunofluorescent staining for class III beta-tubulin (TUJ1) and Peripherin. Neurites that passed through the microchannel originated from PNS neurons, which was confirmed by the expression of TUJ1 and Peripherin (arrowheads) 48 days after cell plating. (E) PNS neurites around the microtunnels 30 days after cell plating. Two different regions, indicated in the diagram, are shown at a high magnification. PNS neurites gathered together and made bundles before and during entering the tunnels. Scale bar: 100 μm.

PNS neurons re-extend their neurites into peripheral tissues, including internal organs and limbs, in response to neuronal injury. The neurite re-innervates target organs and the CNS to re-establish bidirectional signals. However, the process of re-connection between the PNS and CNS is not fully understood yet. Therefore, we developed an *in vitro* cell culture device to image and understand the underlying mechanisms of neuronal wiring. Using PDMS, we fabricated a culture chamber with 2 separate wells and tunnels as outlined in Fig 3. Schematic image of the fabricated culture chambers is also shown in Fig 4A. Importantly, no cross-contamination must occur between the wells, and the tunnels need to allow only the growth of extending neurites to the other side. Therefore, connecting tunnels were designed with a height of 5 μm to exclude neuronal cell bodies from the microtunnels [12,15]. After PNS and CNS neurons were fully differentiated, each type of neuron was collected and re-plated in separate wells of the culture chamber. In order to monitor the cell bodies and neurites of each neuron type, cells were labeled with different dyes before re-plating. During co-culture experiments, neurites entered the microtunnels, but the cell bodies of neurons were excluded (Fig 4B). After 2 weeks of co-culture, neurites reached the opposing well and made neuronal connections. Interestingly, mostly the neurites of PNS neurons crossed the microtunnels (Fig 4C). In contrast, only a few CNS neurites passed through the microtunnels. To confirm these observations, we performed TUJ1 and Peripherin immunostaing, which showed that neurites were double-positive for TUJ1 and Peripherin (Fig 4D). PNS neurites that passed through the microtunnels extended at a rate of approximately 0.2 mm/day during the 2 weeks of co-culture. This rate of growth was lower than that *in vivo* [21,22]. Furthermore, the morphology of PNS neurites suggested that neurites gathered together and made bundles before and during entering the tunnels (Fig 4E), and these bundles passed through the microtunnels together.

**Fig 5:**
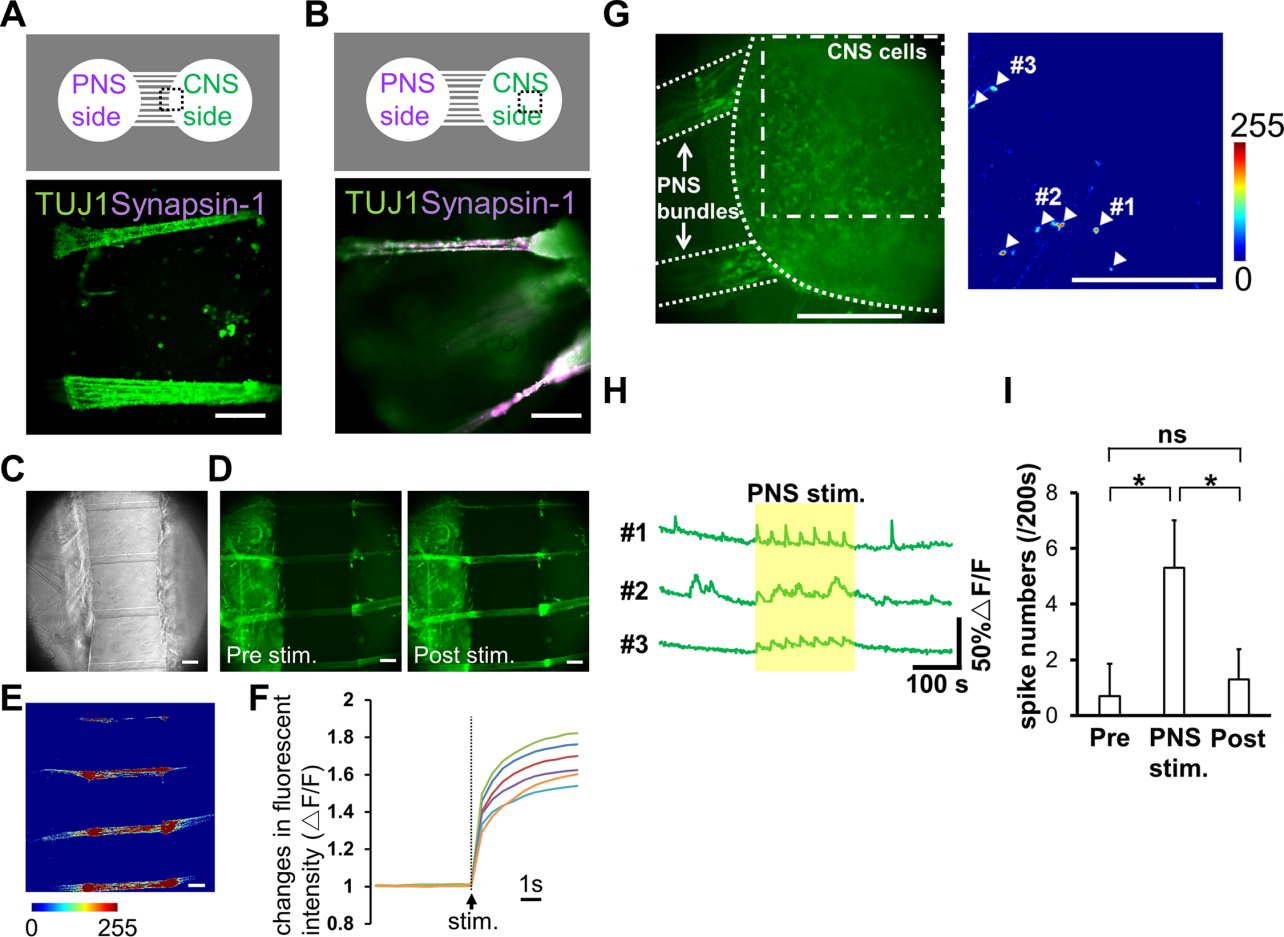
Structural and functional analysis of co-cultured neuronal networks. (A, B) Immunofluorescence images of co-cultured neurons 48 days after cell plating (bottom panel). Different regions indicated in the diagram are shown (top panel). Synapsin-1 staining was detected in the chamber with central nervous system (CNS) neurons, but not in the microtunnels. (C, D) Phase-contrast images of cells and the fluorescence images of a calcium probe on day 39. Fluorescence images before (pre stim) and 1 s after applying the electrical stimulation (post stim) are shown. Fluorescence was increased after the stimulus (arrowheads). (E) Calcium response after the stimulation normalized to the signal before the stimulation. The color bar shows fluorescence intensity. Neurites in the microtunnels were induced with electrical stimulation. (F) Kinetics of calcium transient onset during applying the electrical stimulation. Data of 6 peripheral nervous system (PNS) bundles were considered. (G) Identification of CNS neurons that responded to PNS bundle stimulation. Left, a co-culture sample at day 29 labelled with fluo-4. Right, normalized calcium response in CNS neurons following PNS bundle stimulation (dot-dashed line). The color bar indicates fluorescence intensity. White arrowheads identify CNS neurons that exhibited a calcium response to PNS bundle stimulation. (H) Kinetic plots of calcium transients in CNS neurons represented in panel G. The region highlighted by the yellow rectangle indicates the period of electrical stimulation of PNS bundles. (I) The effect of PNS bundle stimulation on the activity of CNS neurons. Calcium spiking in CNS neurons was averaged pre-, during and post-PNS bundle stimulation. Values are reported as mean±S.D. (*n* = 20 neurons from 3 samples). Statistical analyses using an unpaired t-test are shown here (ns not significant, * *p* < 0.001; Scale bar: 100 μm).

Additionally, we examined whether the connected neurons constructed a neuronal network. To reveal the structure of connection more closely, we evaluated Synapsin-1 expression at the locations of neurite arrival in the CNS well. Synapsin-1 is localized in synaptic vesicles, thus its presence is suggesting axon/neurite interactions. In the well containing CNS neurons, TUJ1-positive PNS bundles integrated with aggregated CNS neurons, as Synapsin-1 was detected in the terminal of the bundles, not in the microtunnels (Fig 5A and 5B). These data indicate that iPS cell-derived PNS and CNS neurons are able to connect with each other and make a neuronal network in our culture device. In addition, a functional assay was carried out using calcium imaging. Preliminarily studies demonstrated that differentiated CNS neurons exhibit spontaneous activity with kinetics typical of neuronal calcium transients (a rapid onset and decay occurring within 10 s, Fig 5H). Alternatively, PNS neurons rarely exhibited spontaneous activity but did respond to the TRPM8 agonist menthol and the TRPV1 agonist capsaicin (data not shown, [23]), which classically activate peripheral afferent neurons. To monitor the responsiveness of PNS bundles to external stimuli, electrical stimulation was applied to the cells with an electric stimulator. Fig 5C and 5D show phase-contrast and fluorescent images around the microtunnel region on day 39. Fluorescence images show the amount of intracellular calcium before and 1 s after applying the electrical pulse. Fig 5E shows normalized differences between signals before and after applying the electrical stimulation (each shown individually in Fig 5D). Electrical pulses elevated the intracellular calcium concentrations of PNS bundles. Kinetics of calcium transient onset before and after the electrical stimulation in 6 PNS bundles from different samples are summarized in Fig 5F. These data indicate that the PNS bundles were functional and could be activated artificially by external stimuli. We then analyzed calcium activity in CNS neurons during electrical stimulation of putatively networked bundles from PNS neurons. An electrical pulse was applied 7 times to bundles from PNS neurons with an interval of 30 s (approximate electrical stimulation period was 200 s in total). Calcium activity was first measured in the bundles and then fluorescence intensity changes were measured in the central regions of CNS wells (Fig 5G). Traces of calcium activity in positively identified CNS neurons at 600 s are shown in Fig 5H. As expected, calcium activity in positively identified CNS neurons was only observed following the axonal bundle stimulation. In conjunction with the statistical data presented in Fig 5I, our data confirm the presence of functional cell-cell interaction between differentiated PNS and CNS neurons. Thus, our culture device could serve as a promising tool to culture different types of neurons and to analyze their interactions.

### Co-culture of PNS neurons and cardiomyocytes in the fabricated culture device

**Fig 6:**
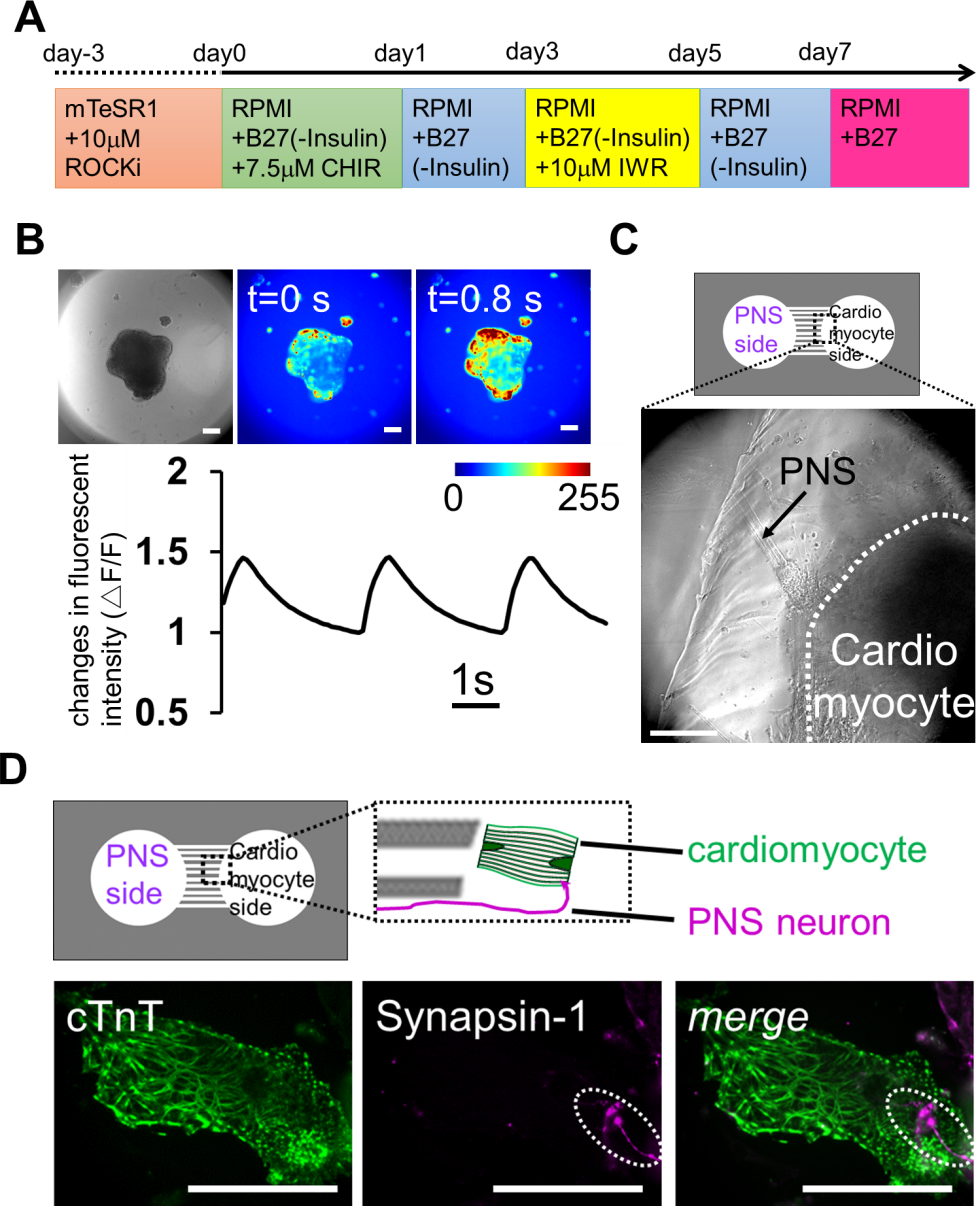
Reconstruction of neuronal networks innervating the heart using iPS cells co-cultured in a microfabricated device. (A) Schematic diagram of the differentiation protocol for iPS cell-derived cardiomyocytes. (B) Calcium imaging of cardiomyocyte-aggregates on day 16. Contracting embryonic bodies (EBs) showed sustained calcium dynamics. The color bar shows fluorescence intensity, and the change in fluorescence signals confirms that these cell-aggregates contained cardiomyocytes. The graph is showing the kinetics of calcium transients during the recording. (C) Co-culture of peripheral nervous system (PNS) neurons and cardiomyocytes on day 44 after plating PNS neurons. PNS-derived bundles extended from left chamber (arrow) and reached a cardiomyocyte-aggregate, which was in the right chamber (within white dash line). (D) Immunostaining for the cardiomyocyte marker cTnT and the synaptic vesicle marker Synapsin-1 on day 24 after plating PNS neurons. Top, the positional relationship of the axon from PNS neurons and cardiomyocyte. Bottom, localization of Synapsin-1 on a cardiomyocyte (dot line region). Scale bar: 100 μm.

Next, we constructed another type of co-culture system using PNS neurons and cardiomyocytes derived from iPS cells. The heart beats autonomously throughout life, but it is also regulated by neuronal stimuli [24,25]. Cardiomyocytes are innervated by PNS neurons, which regulate the beating rate of the heart. Thus, preparing an *in vitro* co-culture system of PNS neurons and cardiomyocytes, both differentiated from same human iPS cells, would be a promising model system for investigating the mechanisms that regulate heart beating, and for drug discovery in cardiovascular diseases associated with peripheral neuropathy. Therefore, we attempted to establish a co-culture using PNS neurons and cardiomyocytes derived from human iPS cells. Following the cell induction method shown in Fig 6A, the EBs of human iPS cells were differentiated into cardiomyocytes. After 2 weeks *in vitro*, the cell aggregates started to contract autonomously (S1 Movie). We also checked the cardiomyocyte beats with calcium imaging, which revealed that almost all cells exhibited calcium oscillations (Fig 6B and S2 Movie).

To establish the compartmentalized co-culture system quickly, PNS neurons were first re-plated in the left side of the chamber and were cultured for two weeks. After we confirmed that the PNS bundles passed through the microchannels, differentiated contracting cardiomyocyte aggregates were isolated and transferred in the right side of the chamber. On the right side of the chamber, the bundles innervated the contracting aggregates (Fig 6C and S3 Movie). To confirm the formation of an apparent connection between PNS neurons and cardiomyocytes, we probed co-culture samples for expression of cTnT and syanpsin-1 in co-culture samples. cTnT expression indicated the presence of mature cardiomyocytes in the well, and PNS-derived Synapsin-1 was co-localized on these cells (Fig 6D). These data indicate the formation of a neuromascular connection between mature cardiomyocytes and PNS neurons in the PDMS devices. These results suggest that our PDMS chamber device is also useful for establishing a connection between PNS neurons and cardiomyocytes and for examining how PNS neurons control cardiomyocyte functions in response to various neuronal stimuli.

## Discussion

Here, we reported the manufacturing of a co-culturing device, which was used for constructing neuronal networks between PNS and CNS neurons derived from iPS cells. The compartmentalized device made from PDMS could be a useful experimental platform for monitoring neurite/cell interactions in various cell types, for screening chemicals, or to investigate the effectiveness or safety of drugs.

Neurites passing through the microtunnels were extended primarily from the PNS neurons towards the chamber containing the CNS neurons. These observations are consistent with those obtained *in vivo*. It is well known that PNS neurons can extend their axons faster and more far than CNS neurons do. However, the growth rate of PNS axons was approximately 0.2 mm/day in our experiments, which was lower than that in humans (1 mm/day) based on clinical studies [21,22]. A possible explanation for this difference might be that growing conditions are different *in vivo* and *in vitro*. Furthermore, PNS bundles in the CNS chamber were exposed to CNS culture medium, which did not contain NGF or NT-3. NGF and NT-3 have been reported to support peripheral nerve regeneration [26,27]. Thus, the absence of these growth factors might affect the growth of PNS bundles. Lack of supportive cells in the PNS chamber might also explain this difference in neurite growing. Schwann cells, which support and myelinate PNS neurons, emerge from human PSCs within one month [28,29]. However, mature astrocytes and oligodendrocytes in CNS require an induction for 2–3 months [30–32] compared with neurons that require one month from human PSCs. Thus, the cellular environment providing support for neurons might not be present in the CNS chamber. Optimizing co-culture conditions to enhance neurite growth should be considered in further investigations. Furthermore, our device might be useful for identifying chemical compounds and growth factors that modulate neurite growth, especially if we can reconstruct the outgrowth of defected neurites, such as those derived of patients with neuropathy.

Our microtunnel structures only allow the isolation axons, but not dendrites, from their cell bodies [12,15]. Hence, PNS bundles originating from PNS neurons, which enter the CNS chamber, are thought to be axons. Accordingly, we did not find synapses within the bundles themselves (Fig 5A). Furthermore, PNS bundles exhibited reproducible evoked responses to electrical stimulations. These functions of PNS cells *in vitro* are consistent with those described in a previous study [14]. As these dynamics are easy to regulate, the system might be suitable for investigating neuronal signal-based inter-organ communication *in vitro*.

Currently, dopaminergic neurons [33], motor neurons [34], and/or other specific types of cells from various organs [35] can be differentiated from human iPS or ES cells. In addition, direct cell conversion techniques are useful for obtaining a purified population of cells [36,37]. Our *in vitro* co-culture model system is suitable for examining morphological and molecular mechanisms underlying tissue development, homeostasis, and diseases using specific types of neurons.

## Conclusions

We co-cultured PNS and CNS neurons derived from human iPS cells in a microfabricated device. We validate methods and protocols for future research on the integration of PNS and CNS neurons to form network structures. In addition, we showed that the co-culture device can be applied for the integration of PNS neurons and other types of cells, such as cardiomyocytes. These data suggest that the compartmentalized PDMS co-culture device is a promising tool for investigating the network-wide integration of PNS neurons, and might help to understand the details of neuronal network mechanisms and the underlying functional mechanisms in normal and pathological conditions.

## Supporting Information

S1 Fig Characterization of human PNS neurons. Immunostaining of peripheral nervous system (PNS) neurons at 15 day showed expression of neural crest marker P75NTR (A) and HNK-1 (B) together with Peripherin, indicating that these neurons were derived from neural crest lineage. Scale bar: 100 μm.
(TIF)

S1 Movie Contracting cardiomyocytes derived from human iPS cells on day 16. Regions (1.78 mm × 1 mm) were captured in real time.
(WMV)

S2 Movie Calcium imaging of the contracting cardiomyocytes on day 16. Regions (1 mm × 1 mm) were captured in real time.
(WMV)

S3 Movie Calcium imaging of co-cultured PNS neurons and cardiomyocytes in the device 44 days after the plating of PNS neurons. 1 mm × 1 mm regions were captured. Speed of the movie is 3 times faster than the real time.
(WMV)
