# Supplementary figures and images for: In Vitro Reconstruction of Neuronal Networks Derived from Human iPS Cells Using Microfabricated Devices
Source: PLoS One. 2016 Feb 5;11(2):e0148559. doi: 10.1371/journal.pone.0148559 (PMC4744060; doi:10.1371/journal.pone.0148559)

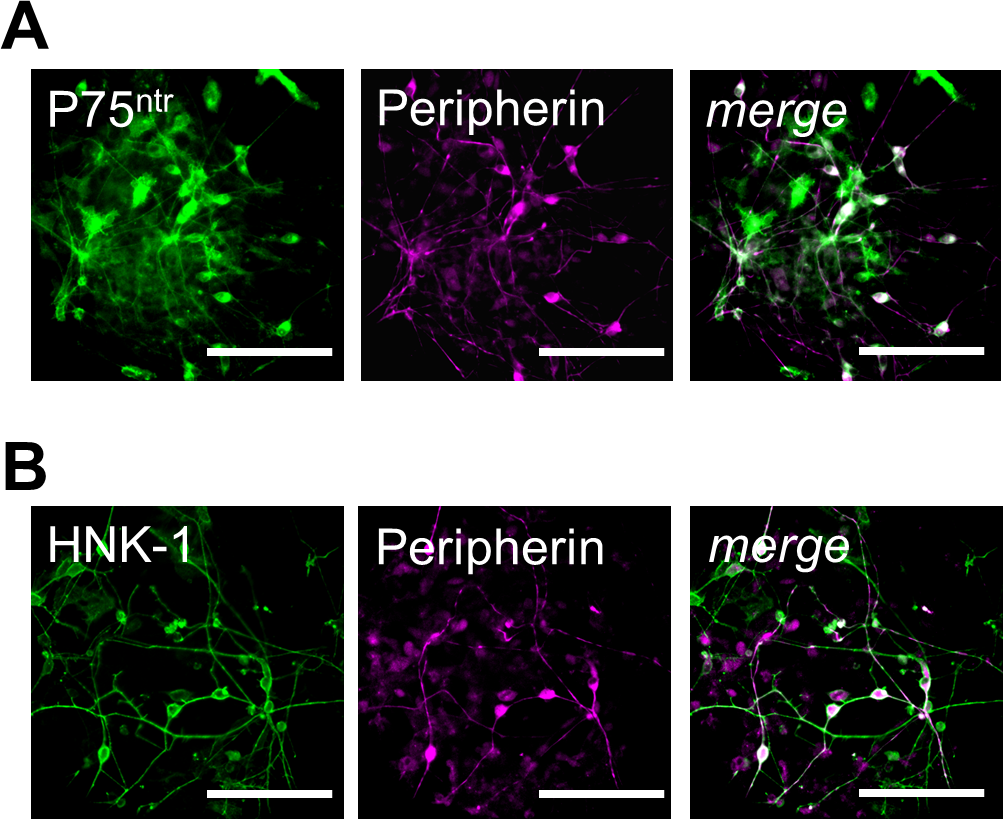

Supplement: S1 Fig — Immunostaining of peripheral nervous system (PNS) neurons at 15 day showed expression of neural crest marker P75NTR (A) and HNK-1 (B) together with Peripherin, indicating that these neurons were derived from neural crest lineage. Scale bar: 100 μm. (TIF) [file pone.0148559.s001.tif]
